# Canine respiratory coronavirus employs caveolin-1-mediated pathway for internalization to HRT-18G cells

**DOI:** 10.1186/s13567-018-0551-9

**Published:** 2018-07-03

**Authors:** Artur Szczepanski, Katarzyna Owczarek, Aleksandra Milewska, Zbigniew Baster, Zenon Rajfur, Judy A. Mitchell, Krzysztof Pyrc

**Affiliations:** 1 Virogenetics, Malopolska Centre of Biotechnology, Jagiellonian University, Krakow, Poland; 2 Faculty of Biochemistry, Biophysics and Biotechnology, Jagiellonian University, Krakow, Poland; 3 Institute of Physics, Faculty of Physics, Astronomy and Applied Computer Sciences, Jagiellonian University, Lojasiewicza 11, 30-348 Krakow, Poland; 4 Department of Pathology and Pathogen Biology, The Royal Veterinary College, Hatfield, Hertfordshire AL9 7TA UK

## Abstract

Canine respiratory coronavirus (CRCoV), identified in 2003, is a member of the *Coronaviridae* family. The virus is a betacoronavirus and a close relative of human coronavirus OC43 and bovine coronavirus. Here, we examined entry of CRCoV into human rectal tumor cells (HRT-18G cell line) by analyzing co-localization of single virus particles with cellular markers in the presence or absence of chemical inhibitors of pathways potentially involved in virus entry. We also targeted these pathways using siRNA. The results show that the virus hijacks caveolin-dependent endocytosis to enter cells via endocytic internalization.

**Electronic supplementary material:**

The online version of this article (10.1186/s13567-018-0551-9) contains supplementary material, which is available to authorized users.

## Introduction

Coronaviruses are enveloped, single-stranded, positive-sense RNA viruses belonging to the family *Coronaviridae* within the order *Nidovirales* [1]. Based on its properties, this family can be divided into four distinct genus: alpha, beta, delta, and gamma. Coronaviruses infect a wide variety of birds and mammals, including humans, livestock, and companion animals [1–3]. Human coronaviruses (HCoVs) are associated mainly with relatively mild upper and lower respiratory tract disease; however, emergence of severe acute respiratory syndrome coronavirus (SARS-CoV) in the winter of 2002–2003 in China, and more recently Middle East respiratory syndrome coronavirus (MERS-CoV) in the Middle East, demonstrates the potential threat posed by zoonotic coronaviruses [2–4].

Canine respiratory coronavirus (CRCoV) was first identified in 2003 in samples obtained from the respiratory tracts of dogs with canine infectious respiratory disease (CIRD; also known as kennel cough) that were housed in animal shelters in the United Kingdom [5]. CIRD is a contagious disease with high morbidity but low mortality; it usually occurs in densely housed dog populations (e.g., rehoming centers, veterinary hospitals). Characterized by a dry, hacking cough, the disease is generally mild and self-limiting. However, it can progress to a potentially fatal bronchopneumonia [6, 7]. CIRD is considered a complex infection, with a multifactorial etiology in which a number of organisms (including *Bordetella bronchiseptica*, canine parainfluenza virus, canine adenovirus type 1 and 2, canine herpesvirus, *Mycoplasma* spp., canine pneumovirus, and influenza viruses) are involved [6, 8]. It is believed that CRCoV plays a role in the early stages of CIRD by limiting ciliary clearance of the upper airways. Consequently, infection leads to reduced respiratory clearance and sensitization to secondary infections [5–7].

CRCoV is closely related to two other betacoronaviruses, bovine coronavirus (BCoV) and HCoV-OC43 (97.3% nucleotide identity in the spike gene for BCoV and 96.9% for OC43 as reported by Erles et al. [5]), but is clearly distinct from Canine Enteric Coronavirus (CECoV, previously known as Canine Coronavirus) [5, 7]. CRCoV is a difficult pathogen to work with because the only confirmed susceptible cell line is a human rectal tumor cell line (HRT-18) and its derivative HRT-18G. No canine cell line supports replication of the virus. Furthermore, CRCoV does not produce a cytopathic effect in HRT-18 cells [8].

To initiate infection, enveloped viruses fuse with host cell membrane prior to delivering genetic material. This process may occur at the cell surface (e.g., human immunodeficiency virus, herpes simplex virus); otherwise prior internalization is required [2, 9]. To enter the cell, viruses hijack a number of different endocytic pathways, including macropinocytosis and clathrin-mediated, caveolin-mediated, and clathrin- and caveolin-independent routes [2, 9, 10]. For example, SARS-CoV uses clathrin-dependent, lipid raft-mediated, and clathrin- and caveolae-independent entry pathways [2, 11–13]. In addition, feline infectious peritonitis virus (FIPV) uses clathrin- and caveolin-independent endocytic routes [14], whereas HCoV-229E uses caveolae-dependent endocytosis [15]. Furthermore, some human respiratory coronaviruses may utilize protease activation to modulate the route of entry [16–18]. Generally, within each of these endocytic pathways, vesicles are formed through interaction of certain protein networks. Early vesicles provide a starting point for trafficking, which leads to endosome maturation and allows sorting of incoming cargo [19, 20]. Some internalized vesicles are recycled back to the cell surface, while others are converted, for example to lysosomes. Sorting of cargo is regulated by Rab GTPases, which serve as molecular hallmarks of different routes [19–21].

Here, we studied internalization of CRCoV into HRT-18G cells. The results clearly demonstrated that CRCoV entry into HRT-18G cells requires endocytic internalization prior to membrane fusion, a process that requires caveolin-1 and dynamin. Furthermore, fusion of the viral and cellular membranes occurs before the endosome progresses to the late phase.

## Materials and methods

### Cells and viruses

HRT-18G (ATCC CRL-11663) cells, derivative of HRT-18 (ATCC CCL-244, ileocecal colorectal adenocarcinoma) were maintained in Dulbecco’s MEM (Life Technologies, Poland) supplemented with 3% heat-inactivated fetal bovine serum (Life Technologies), penicillin (100 U/mL), streptomycin (100 μg/mL), and ciprofloxacin (5 μg/mL). Cells were cultured at 37 °C under 5% CO_2_. Virus stock of canine respiratory coronavirus strain 4182 was prepared by infecting HRT-18G cells monolayers and collecting supernatant 5 days post-infection (pi). Obtained stock was aliquoted and stored at −80 °C. The control from mock-infected cells was prepared in the same manner. Virus yield was estimated by titration on confluent HRT-18G cells according to the method of Reed and Muench [22]. As CPE is not visible, cells were infected at 37 °C for 5 days, fixed and immunostained to detect virus-infected cells. For co-localization studies, stocks were concentrated using Amicon Ultra Centrifugal Filters (Merck, 10-kDa cutoff), aliquoted, and stored at −80 °C.

### Chemical inhibitors

DMEM supplemented with 100 U/mL penicillin, 100 μg/mL streptomycin and one of the following chemical inhibitors: 1-aminoadamantane (100 µM, AMTD, Sigma-Aldrich, Poland), Pitstop (10 µM, Abcam), chlorpromazine (1.5 µM, Sigma-Aldrich), filipin III (2 μg/mL, Sigma-Aldrich), nystatin (50 µg/mL, Sigma-Aldrich), methyl-β-cyclodextrin (5 mM, MβCD, Sigma-Aldrich), 5-(*N*-ethyl-*N*-isopropyl)amiloride (10 µM, EIPA, Sigma-Aldrich), 1,1′-Dithiobis-2-naphthalenol (20 µM, IPA-3, Sigma-Aldrich), wortmannin (5 µM, Calbiochem), dynasore (80 µM, Abcam), iminodyn 22 (25 µM, Abcam), mitmab (5 µM, Abcam), ammonium chloride (50 mM, NH_4_Cl, Bioshop), bafilomycin A1 (10 nM, Sigma-Aldrich), cytochalasin D (10 µM, Sigma-Aldrich), jasplakinolide (1.5 µM, Calbiochem), nocodazole (0.5 μM, Sigma-Aldrich), cell permeable Rho inhibitor (1 µg/mL, CT04 Cytoskeleton Inc.), *N*^6^-[2-[[4-(Diethylamino)-1-methylbutyl]amino]-6-methyl-4-pyrimidinyl]-2-methyl-4,6-quinolinediamine trihydrochloride (100 μM NSC23766), (*R*)-(+)-*trans*-4-(1-Aminoethyl)-*N*-(4-Pyridyl)cyclohexanecarboxamide dihydrochloride (10 μM, Y27632 Sigma-Aldrich), Decanoyl-Arg-Val-Lys-Arg-chloromethyl ketone (5–100 μM, Santa Cruz Biotechnology) were used to pretreat HRT-18G cells for 1 h at 37 °C.

### Inhibition of viral replication

Cells pretreated with chemical inhibitors for 1 h at 37 °C (full list at Additional file 1), were exposed to virus at a 50% tissue culture infectious dose (TCID_50_) of 400 in the presence of inhibitors. Two hours pi cells were washed with PBS twice to remove unbound virus, and medium with fresh inhibitors was added to each well. Five days pi cells were harvested for further analysis. Cell viability was tested at day five pi using XTT based Cell Proliferation Kit (Biological Industries), according to the manufacturer’s instructions.

### FACS analysis

Cells treated with inhibitors were harvested at 5 day pi by trypsinization, centrifuged (5 min, 300 × *g*), washed with PBS, fixed in 4% formalin in PBS (15 min, room temperature (RT)) and permeabilized (0.5% Triton X-100 in PBS, 20 min, RT, Bioshop). Unspecific binding sites were blocked (5% bovine serum albumin (BSA, Bioshop) in PBS, 2 h RT) prior to staining. Further, cells were incubated with anti-coronavirus antibody OC43 strain (1 µg/mL, Merck) for 2 h and with secondary Alexa Fluor 488 goat anti-mouse antibody (5 µg/mL, Thermo Scientific, Poland) for 1 h. Cells were washed with 0.5% Tween-20, resuspended in PBS, and analyzed with flow cytometry (FACSCalibur, Becton Dickinson).

### Confocal microscopy

Cells cultured on coverslips in 6-well plate for 48 h were washed with ice-cold PBS and incubated with concentrated CRCoV at 4 °C for 60 min and subsequently incubated at 37 °C for 0–180 min. Coverslips were washed thrice with PBS, fixed in 4% formalin for 15 min, permeabilized with 0.5% Tween-20 (Bioshop) for 20 min (unless stated otherwise). Unspecific binding sites were blocked using 5% BSA in PBS (4 °C, overnight) prior to staining. For visualization of CRCoV particles anti-coronavirus antibody OC43 strain (1 µg/mL, 2 h, RT, Merck) coupled with goat anti-mouse Alexa Fluor 488 antibody (5 µg/mL, 1 h, RT, Thermo Scientific) was used. To visualize host cell proteins, cells were blocked using 10% FBS in PBS (4 °C, overnight) and incubated (2 h, RT) with one of the following antibodies: caveolin-1 antibody (2 µg/mL, Santa Cruz Biotechnology), clathrin HC antibody (2 µg/mL, Santa Cruz Biotechnology), EEA1 antibody (2 µg/mL, Santa Cruz Biotechnology), Rab 7 antibody (2 µg/mL, Santa Cruz Biotechnology), endophilin B2 antibody (2 µg/mL, Santa Cruz Biotechnology), Rab 11 antibody (2.67 µg/mL, Proteintech), LAMP antibody (10 µg/mL, Thermo Scientific) coupled with secondary goat anti-rabbit Alexa Fluor 546 (10 µg/mL, Thermo Scientific). Signal specificity was verified using isotype control (normal rabbit IgG, normal goat IgG, Santa Cruz Biotechnology). Following this, cells were washed thrice with 0.5% Tween-20 in PBS. Nuclear DNA was stained with 4′,6′-diamidino-2-phenylindole (DAPI, 0.1 μg/mL, Sigma-Aldrich). Stained coverslips were mounted on glass slides in Prolong Diamond medium (Thermo Scientific). Fluorescent images were acquired using Zeiss LSM 710 confocal microscope (Carl Zeiss Microscopy GmbH).

### Inhibitors influence on initial phase of infection

Cells pretreated with chemical inhibitors were incubated with concentrated CRCoV in the presence of inhibitors at 37 °C for 2 h. Two hours pi cells were washed with PBS twice to remove unbound virus, fixed with 4% formalin in PBS (15 min, RT) and permeabilized with 0.5% Tween-20. Unspecific binding sites were blocked using 5% BSA in PBS (4 °C, overnight) prior to staining. CRCoV was visualized using anti-coronavirus antibody OC43 strain (1 µg/mL, 2 h, RT, Merck) coupled with goat anti-mouse Alexa Fluor 488 antibody (5 µg/mL, 1 h, RT, Thermo Scientific). For this study Alexa-Fluor 647 Phalloidin (4 U/mL, 1 h, RT, Thermo Scientific) labeled actin cortex was assumed to indicate cell surface. Nuclear DNA was stained with DAPI (0.1 μg/mL, Sigma-Aldrich) and coverslips were mounted on glass slides in Prolong Diamond medium.

### Post-entry inhibitory effects

Cells were infected with CRCoV at TCID_50_ of 400/mL. After 2 h incubation unbound virus was washed off with PBS and cells were overlaid with culture medium containing chemical inhibitors. Five days pi cells were harvested for further analysis.

### Role of furin during the infection

Cells were infected with CRCoV at TCID_50_ of 400/mL. After 2 h incubation at 37 °C unbound virus was washed off with PBS and cells were overlaid with culture medium containing decanoyl-RVKR-chloromethyl ketone (dec-RVKR-CMK). Four days pi cells were fixed, stained and fluorescent images were acquired.

### siRNA transfection

HRT-18G cells were transfected with 25 pmol of caveolin-1 siRNA (sc-29241, Santa Cruz Biotechnology) or scrambled negative control siRNA (sc-44237, Santa Cruz Biotechnology) using lipofectamine RNAiMAX reagent (Thermo Scientific) according to manufacturer’s protocol. Two consecutive transfections were performed 24 h and 48 h after cell seeding. Subsequently, cells were infected and prepared for imaging.

### Computer analysis

All graphs presented in this work were created using GraphPad Prism 6 software. Significance was estimated using one-way ANOVA with multiple comparisons to virus control. Images obtained from the confocal microscope were deconvolved using AutoQuant X3 and processed in ImageJ Fiji [23]. Co-localization analyses were performed in ImageJ using JACoP plugin [24] where Pearson’s and Manders’ coefficient were calculated for 3D cell reconstructions. The bioinformatics analysis was conducted using arginine and lysine propeptide cleavage sites prediction algorithms ProP 1.0 server [25] using the following sequence data: AAO06124.1 (isolate 4182), ABG78748.1 (isolate 4182), ACX46840.1 (strain K9), AFW97360.1 (strain K37), ACX46860.1 (strain K39) and AQT26498.1 (strain BJ232).

## Results

### CRCoV enters HRT-18G cells via endocytosis

**Figure 1:**
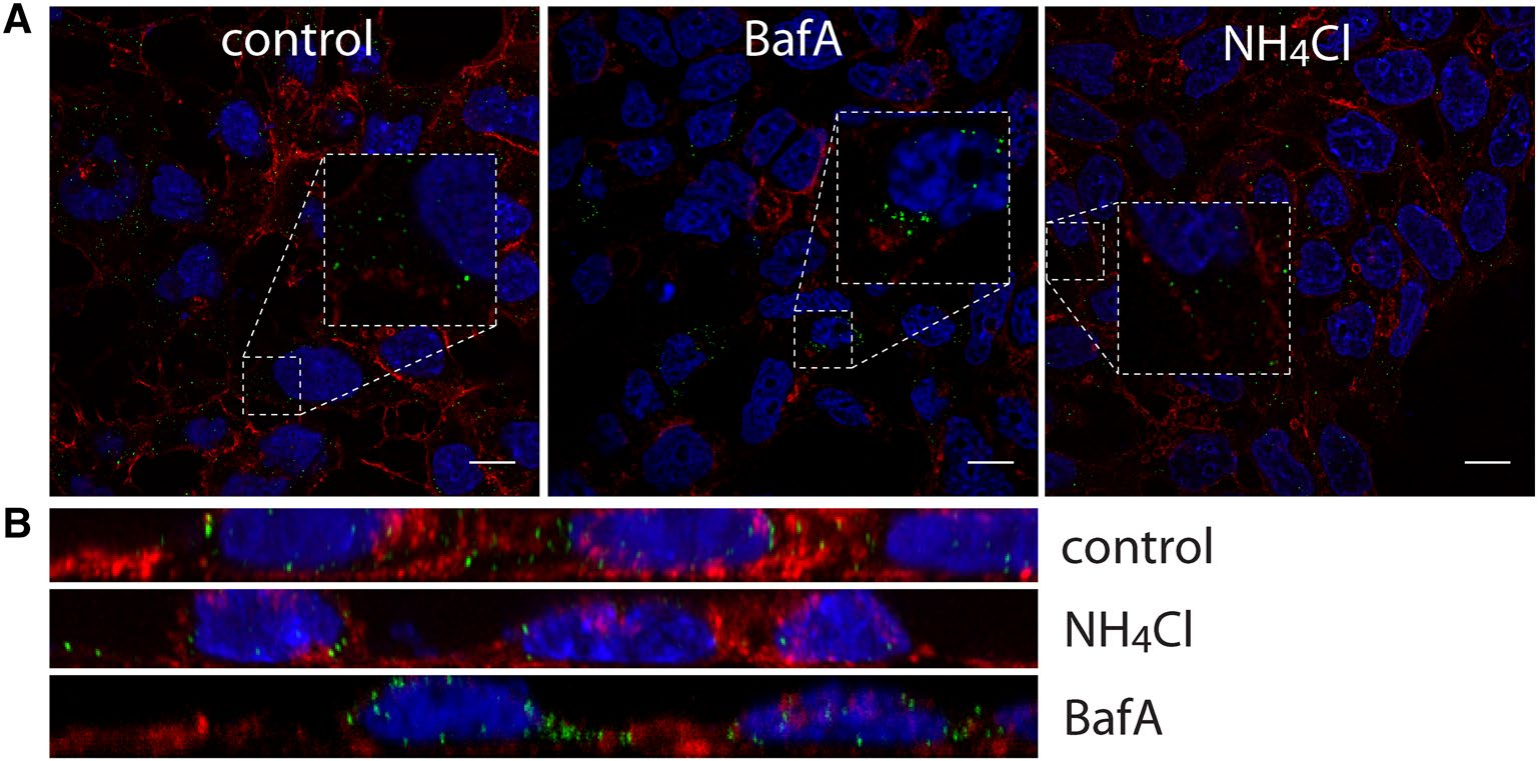
**Effect of endosome alkalization on CRCoV infection. A** Analysis of inhibitors effect on CRCoV entry, cells were pretreated with inhibitors, infected in presence of them and incubated for 1 h before they were washed off and fixed. CRCoV virions are presented in green, blue denotes DNA and red represents actin, each image is a single confocal plane while **B** represents maximum projections of their axial planes. Scale bar 10 µm.

Weak bases such as NH_4_Cl were previously described to hamper endosomal entry of viruses, by preventing pH-dependent activation of the fusion protein, subsequently blocking membrane fusion [26]. Treatment of HRT-18G cells with 50 mM NH_4_Cl inhibited replication of CRCoV (5.9 ± 9.9% of control, Additional file 2), but did not inhibit virus entry into host cells (Figures 1A and B). Bafilomycin A1 inhibits vacuolar H^+^-ATPases and blocks endosome acidification [27]. Similarly, bafilomycin A1 blocked virus replication (93.5% ± 5.2% reduction in number of infected cells, compared to control) (Additional file 2), but not virus entry (Figures 1A and B). Surprisingly, addition of bafilomycin A1 or NH_4_Cl 2 h pi also resulted in decreased virus replication. Similar observations were made for the most of tested inhibitors of intracellular trafficking (Additional file 2). For this reason we decided to use for the analysis only the data on virus entry.

**Figure 2:**
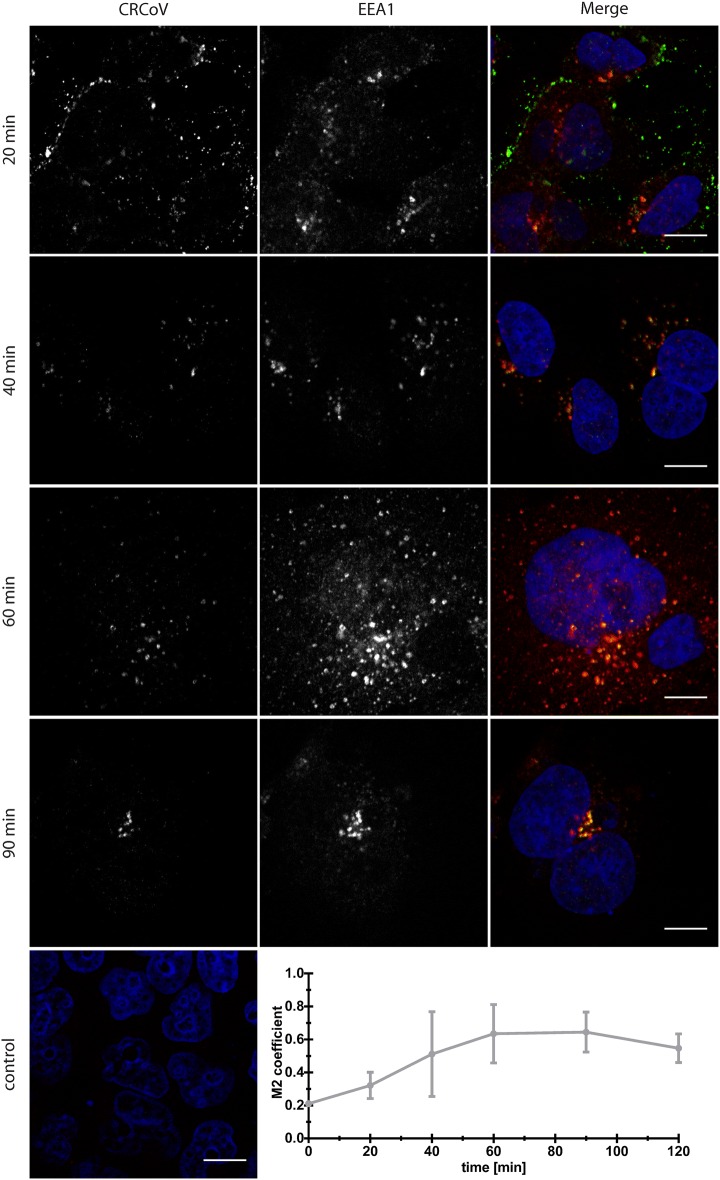
**Co-localization of CRCoV with early endosome marker EEA1.** Virus treated cells were synchronized on ice for 60 min and incubated at 37  °C before they were washed and fixed with 4% paraformaldehyde in PBS. Localization was analyzed by confocal microscopy after performing a double immunofluorescence staining to visualize virus nucleocapsid (left) and early endosomes (middle). Right panel shows merged image with virus presented as green and EEA1 as red. Cell nuclei are shown in blue. Control—negative control, cells incubated with mock sample (green) and stained with isotype antibodies (red). Scale bar 10 µm. Graph presents co-localization change in time.

Consequently, to test whether the virus enters the cell by endocytosis, we examined co-localization of viral particles with early endosome antigen 1 (EEA1) at different times post-infection (pi). Co-localization was clearly visible (Figure 2), peaking at 60–90 min pi (Mander’s coefficient 0.63 ± 0.18 at 60 min and 0.64 ± 0.12 at 90 min pi). No co-localization with the late endosome marker Rab 7, the lysosome marker LAMP1, or the endosome marker Rab 11 was visible at any time (Additional files 3, 4).

**Figure 3:**
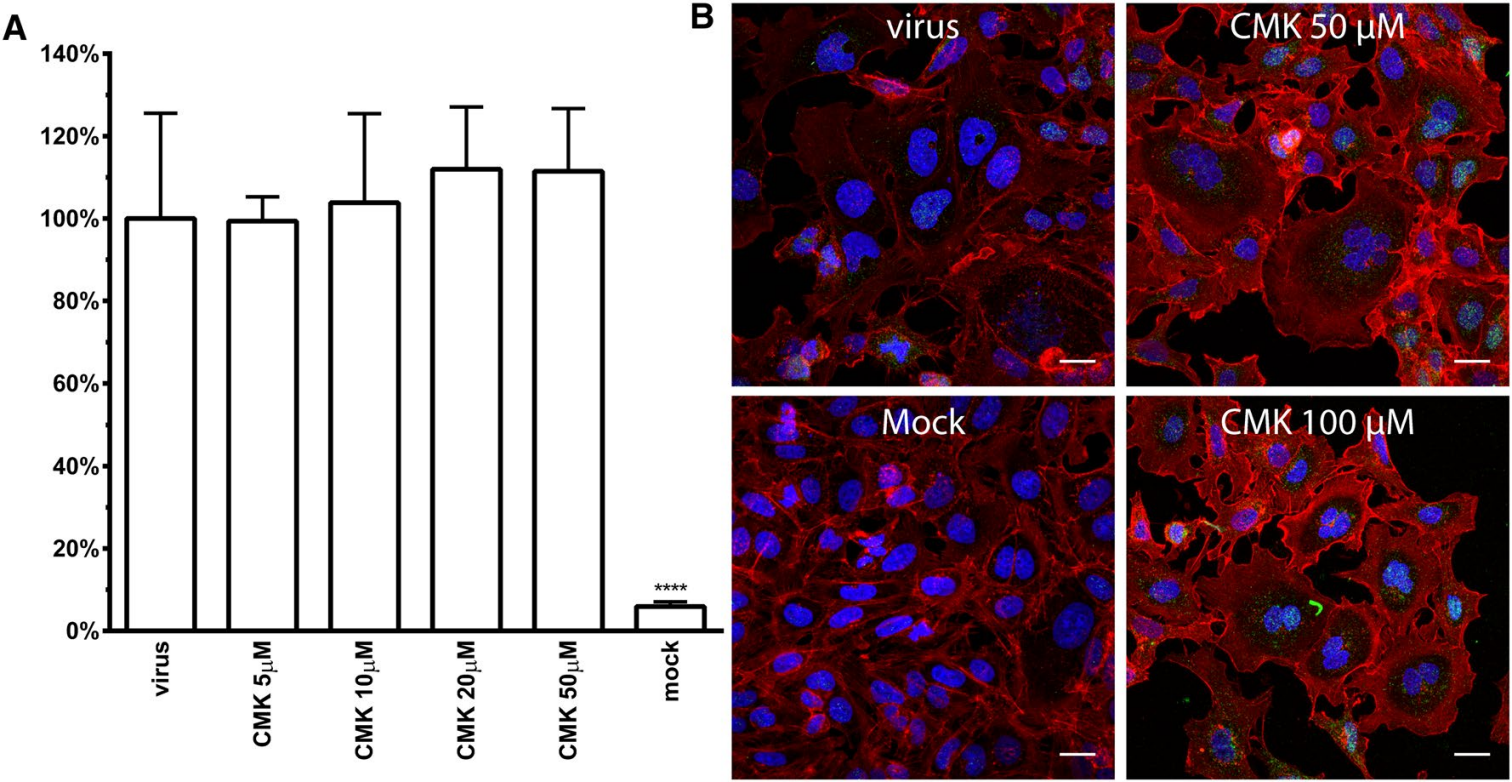
**Effect of furin inhibitor—decanoyl-RVKR-chloromethyl ketone (CMK) on CRCoV replication (A) and cell to cell spread (B). A** Graph shows number of virus positive cells at 4^th^ day pi normalized to control. HRT-18G cells were pretreated with inhibitor before infection and it was present post-infection. **B** 3 h pi cells were overlaid with CMK containing medium and were propagated in this conditions until fixation at 4^th^ day pi. CRCoV virions are presented in green, blue denotes DNA and red represents actin. Scale bar 20 µM.

Obtained results suggest that CRCoV undergoes fusion already in an early endosomal compartment, what may seem contradictory to some previous reports [2, 28–30]. However, some viruses are processed by furin in the producer cell, what makes the processing by cathepsins dispensable [31]. As in silico analysis predicted potential furin cleavage site in CRCoV spike glycoprotein gene (Additional file 5), effect of dec-RVKR-CMK (5–100 µM, Santa Cruz Biotechnology) on virus replication as well as cell-to-cell spread was tested. As visible on Figure 3 no alteration of virus entry was observed.

### Inhibition of CRCoV entry into HRT-18G cells

Next, we used inhibitors of endocytosis to examine the endocytic pathway utilized by CRCoV to enter HRT-18G cell. All inhibitors were used at the highest non-toxic concentration (determined in an XTT assay; data not shown). The validity of the obtained data was verified using positive controls (transferrin, cholera toxin subunit B (CHT_x_B), and dextran), which enter the cell via clathrin-dependent pathway, caveolin-dependent pathway, and macropinocytosis, respectively [9, 32].

**Figure 4:**
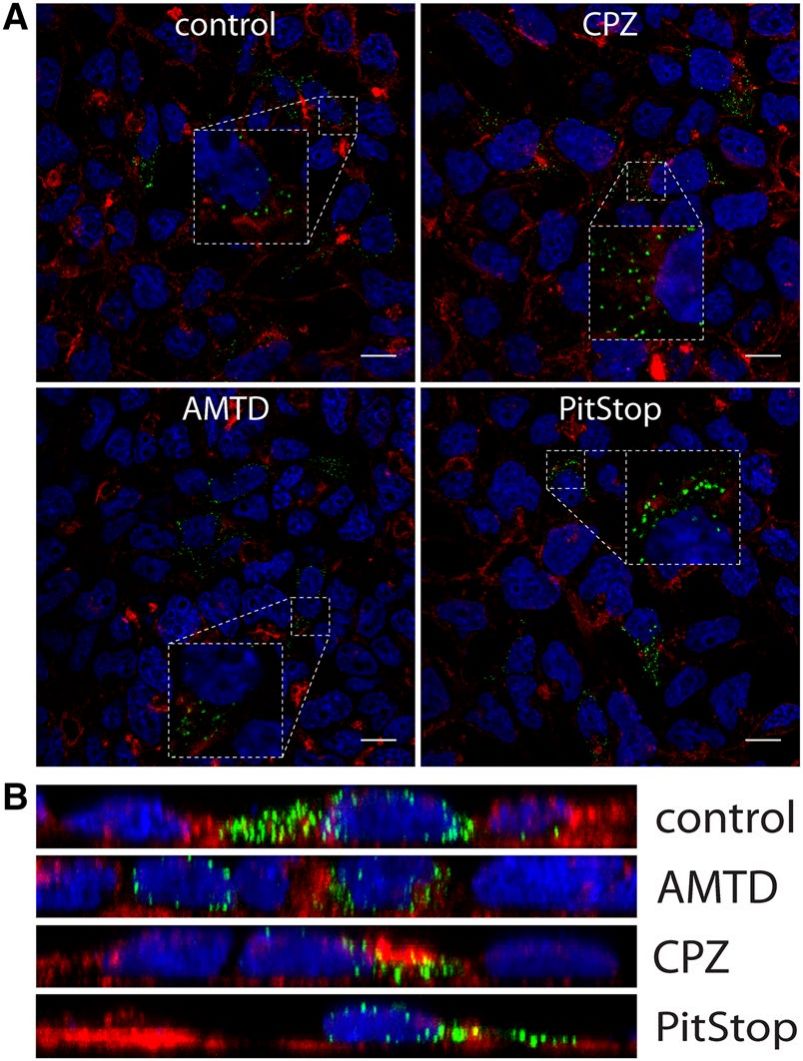
**Effect of clathrin-mediated endocytosis inhibitors on CRCoV infection. A** Analysis of inhibitors effect on CRCoV entry, cells were pretreated with inhibitors, infected in presence of them and incubated for 1 h before they were washed off and fixed. CRCoV virions are presented in green, blue denotes DNA and red represents actin, each image is a single confocal plane while **B** represents maximum projections of their axial planes. Scale bar 10 µm.

To determine whether CRCoV enters HRT-18G cells by clathrin-mediated endocytosis (CME), we treated cells with chlorpromazine, amantadine, or PitStop-2 [33, 34]. None of the compounds affected the virus entry to the cells (Figures 4A and B).

**Figure 5:**
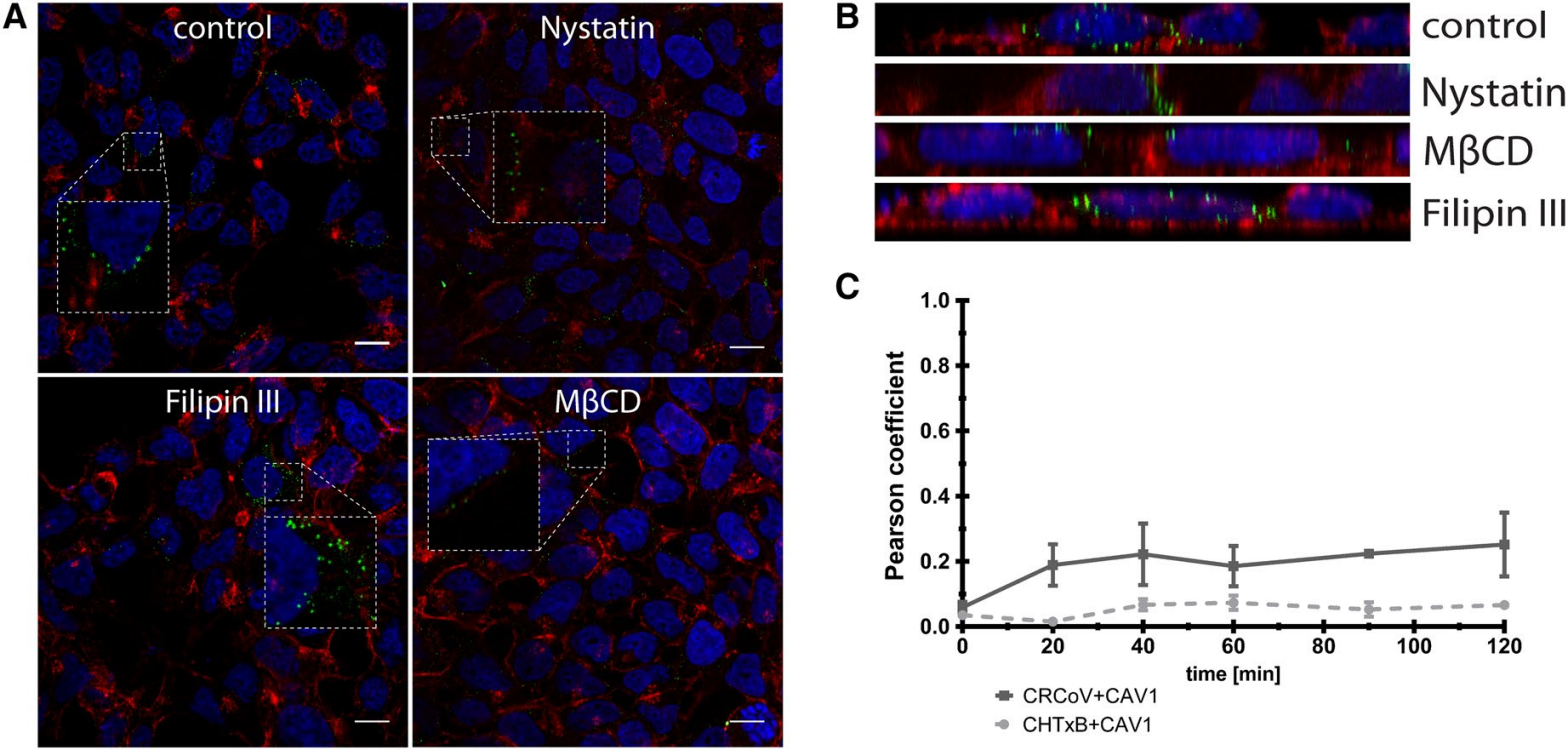
**Effect of caveolin-mediated endocytosis inhibitors on CRCoV. A** Analysis of inhibitors effect on CRCoV infection, cells were pretreated with inhibitors, infected in presence of them and incubated for 1 h before they were washed off and fixed. CRCoV virions are presented in green, blue denotes DNA and red represents actin, each image is a single confocal plane while **B** represents maximum projections of their axial planes. **C** Comparison of CRCoV and CHTxB co-localization with caveolin-1. Scale bar 10 µm.

**Figure 6:**
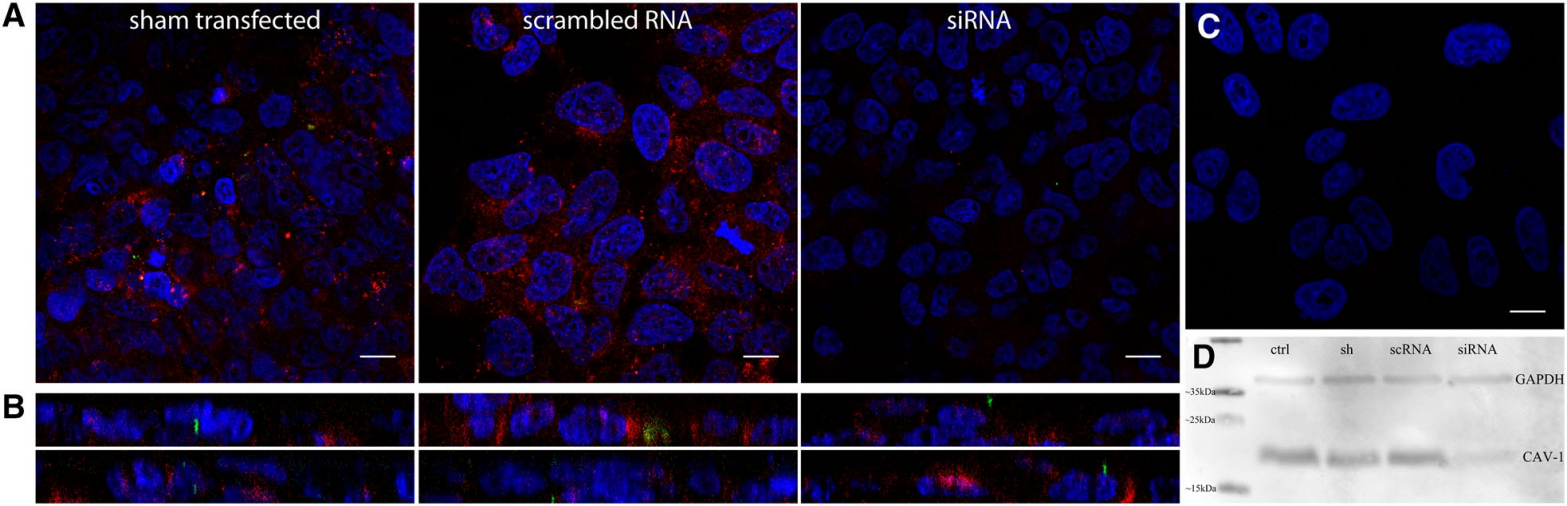
**siRNA treatment.** HRT-18G cells were transfected twice using scrambled negative control siRNA or caveolin-1 siRNA, infected, synchronized on ice for 60 min and incubated at 37  °C for 60 min before they were washed and fixed. CRCoV virions are presented in green, blue denotes DNA and red represents caveolin 1 (**A**) or actin (**B**), each image is a single confocal plane. **C** Negative control for staining. Bar 10 µm **D** Western blot signal of caveolin-1 and GAPDH expression in transfected cells. ctrl: control, sh: sham transfected cells, scRNA: scrambled RNA, siRNA: caveolin-1 siRNA. Scale bar 10 µm.

Caveolae, rich in cholesterol and sphingolipids, are disrupted by sterol-binding agents, as nystatin, filipin, or MβCD. Filipin had no effect on virus entry; neither did it hamper internalization of CHT_x_B, implying ineffectiveness of this inhibitor on HRT-18G cell line. Nystatin and MβCD blocked virus internalization to the cell (Figures 5A and B), suggesting that caveolae are essential during CRCoV internalization. To ensure that the observed effect is not an artifact, caveolin-1 expression was silenced using siRNAs. As shown in Figure 6, depletion of caveolin-1 resulted in reduction in number of virus particles entering the cell.

**Figure 7:**
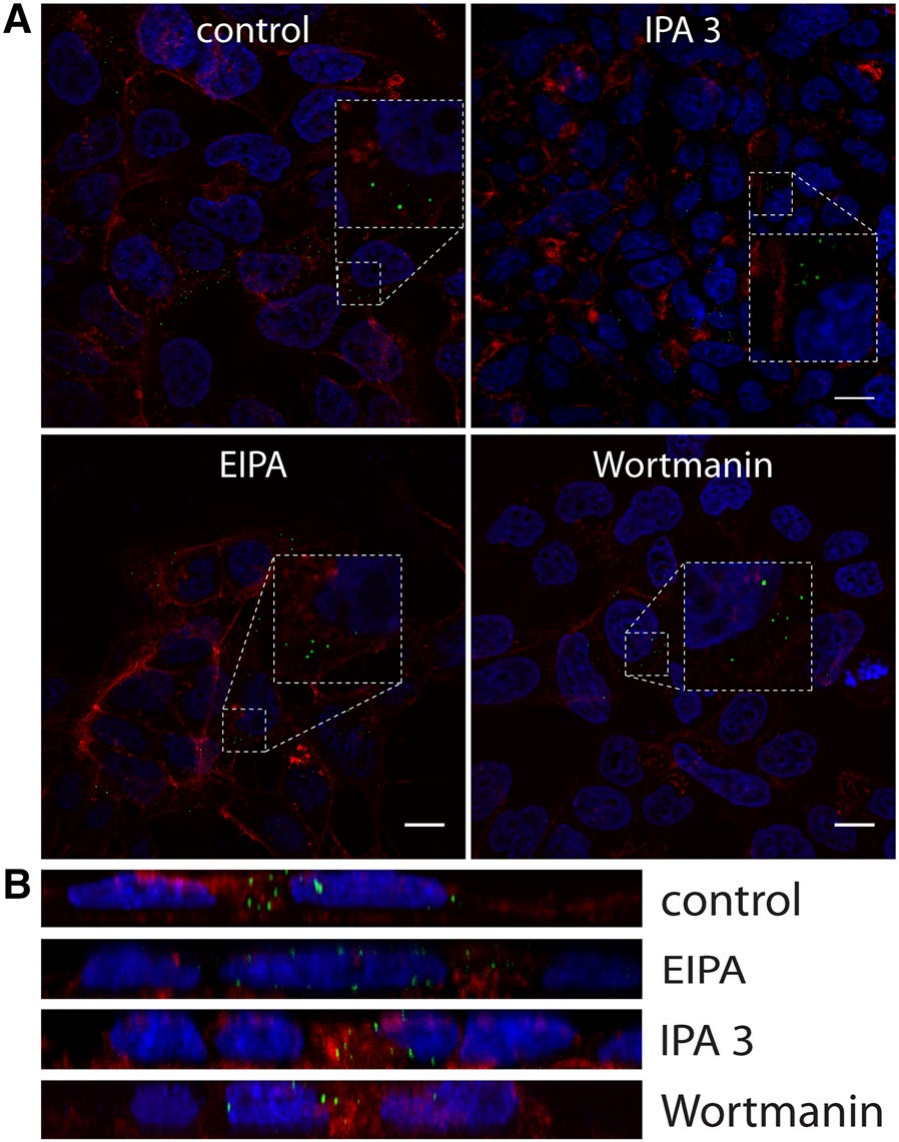
**Effect of macropinocytosis inhibitors on CRCoV infection. A** Analysis of inhibitors effect on CRCoV entry, cells were pretreated with inhibitors, infected in presence of them and incubated for 1 h before they were washed off and fixed. CRCoV virions are presented in green, blue denotes DNA and red represents actin, each image is a single confocal plane while **B** represents maximum projections of their axial planes. Scale bar 10 µm.

To check whether the virus enters cells via macropinocytosis, we treated them with the NA^+^/H^+^ exchanger inhibitor EIPA, the Pak-1 inhibitor IPA-3, and the PI3K inhibitor wortmannin [35]. No effect on virus entry was, however, observed (Figures 7A and B).

### Co-localization of virus particles with markers of endocytic pathways

**Figure 8:**
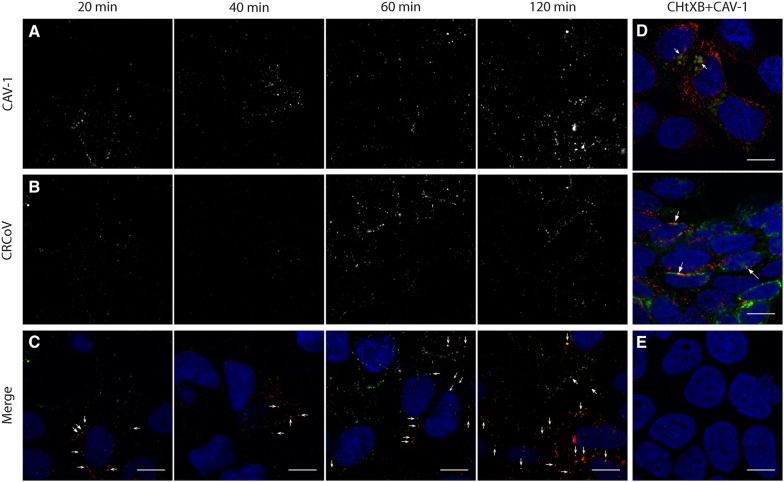
**CRCoV co-localize with caveolin.** Cells treated with virus were synchronized on ice for 60 min and incubated at 37  °C for 20, 40, 60, or 120  min before they were washed and fixed. Caveolin-1 are presented on **A** (red channel) while **B** shows stained virus nucleocapsid protein (green channel). **C** Depicts merged image with marked co-localization. **D** FITC conjugated CHTxB (green) co-localizing with caveolin-1 (red) after 40 (upper) and 60 (lower) min. **E** Negative controls for staining. Scale bar 10 µm.

**Figure 9:**
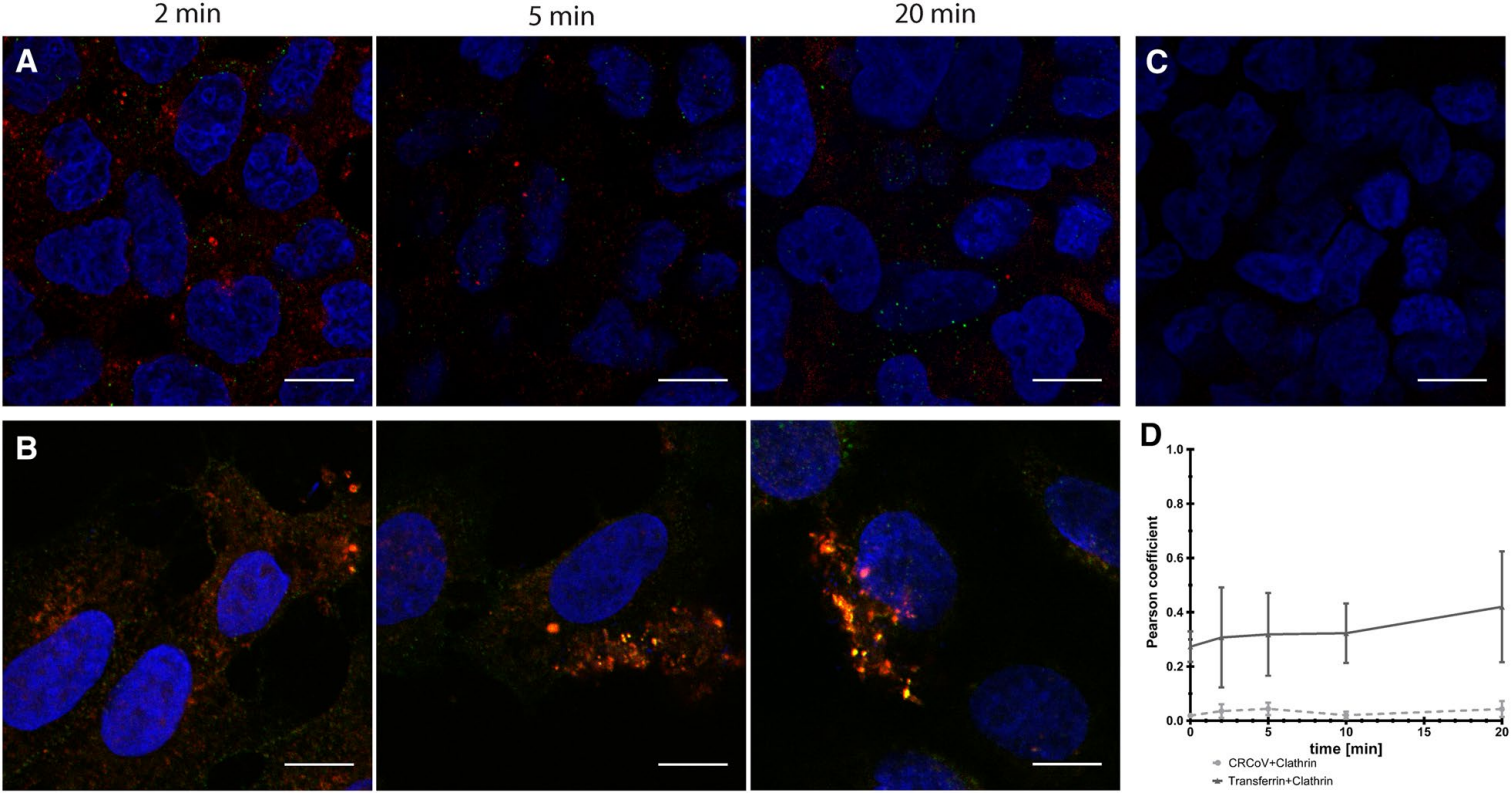
**CRCoV (A) does not co-localize with clathrin in opposite to Alexa Fluor 488 conjugated transferrin (B).** Cells treated with virus or transferrin were synchronized on ice for 60 min and incubated at 37 °C for 2, 5 or 20 min before they were washed and fixed. Clathrin HC are presented in red while stained virus nucleocapsid protein in green. **C** Negative controls for staining. **D** Co-localization changes in time. Scale bar 10 µm.

The above results showed that only compounds that interfere with caveosome formation affect CRCoV entry. However, some reports show that these compounds have off-target and multi-target activity [36–39]. Therefore, to ensure the validity of the obtained data, we examined co-localization of virus particles with markers of different endocytic pathways. Cells were exposed to virus for 0–180 min. As shown in Figure 8, CRCoV co-localized with caveolin at 20 min pi, and this was maintained for up to 120 min pi. Visual assessment and analysis of co-localization coefficients revealed that co-localization was more marked than for CHT_x_B, which utilizes the caveolin-1 mediated pathway to enter cells (Figures 5C and 8D). We also examined whether the virus co-localizes with clathrin (Figure 9) or endophilin (Additional file 6). No co-localization was detected.

### Dynamin is important for CRCoV internalization

**Figure 10:**
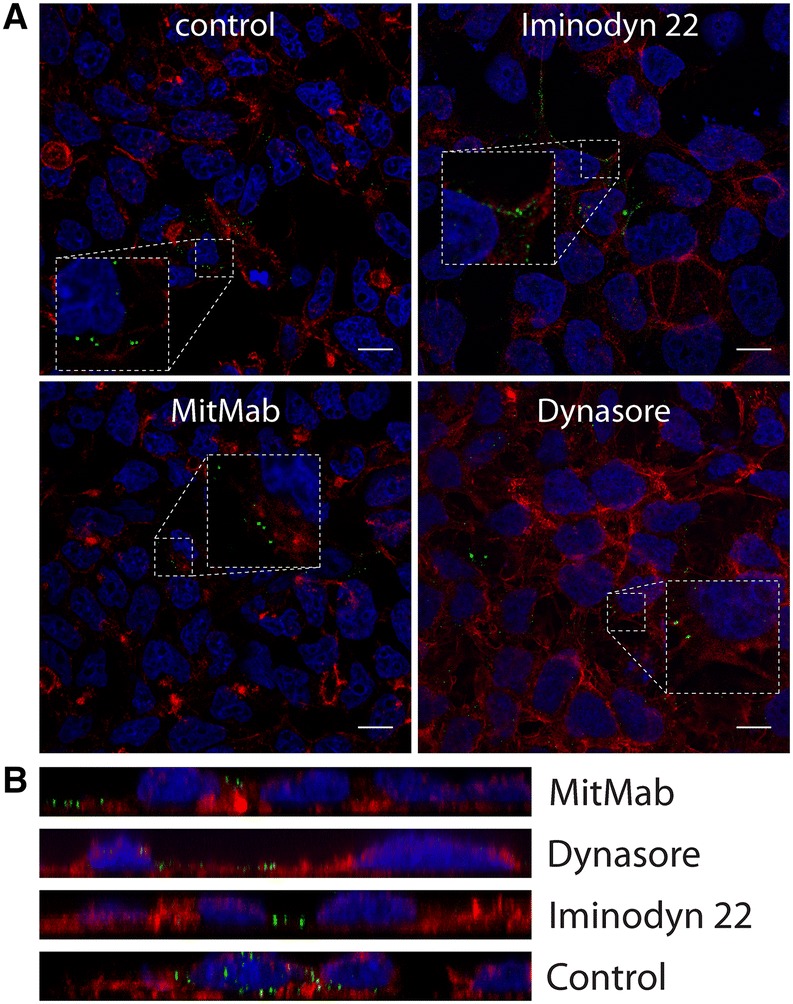
**Vesicle scission—dynamin dependence. A** Analysis of inhibitors effect on CRCoV entry, cells were pretreated with inhibitors, infected in presence of them and incubated for 1 h before they were washed off and fixed. CRCoV virions are presented in green, blue denotes DNA and red represents actin, each image is a single confocal plane while **B** represents maximum projections of their axial planes. Scale bar 10 µm.

Dynamin is essential for several endocytic pathways [14, 40, 41]. Therefore, we asked whether this GTPase plays a role in CRCoV entry into cells. For this, we used three inhibitors of dynamin, which inhibited GTPase activity (dynasore and iminodyn-22) [42, 43] or blocked the lipid binding (MiTMAB) [44]. All three inhibitors affected virus replication: dynasore by 87.8 ± 7.5%, iminodyn-22 by 60.0 ± 1.5%, and MiTMAB by 94.1 ± 3.7% (Additional file 2) when added before infection. What is even more important, their effect was limited when added only post-infection (Additional file 2), suggesting that dynamin is important at early stages of infection. All inhibitors caused a marked reduction in the number of virions entering the cell (Figures 10A and B). Recently, Xu et al. [45] studied JEV entry into the cells and proposed a new model—actin- and dynamin-dependent caveolae-mediated endocytosis. Thus, we analyzed the effect of inhibiting Rho, Rac1, and ROCK kinases on virus infection. Only inhibition of Rac1 (by NSC23766) hampered viral replication showing significantly stronger effect when present prior to infection (7.9% ± 9.3 of control) than after (51.9% ± 3.6 of control) (Additional file 2) implying its involvement in entry process.

### Cytoskeleton

**Figure 11:**
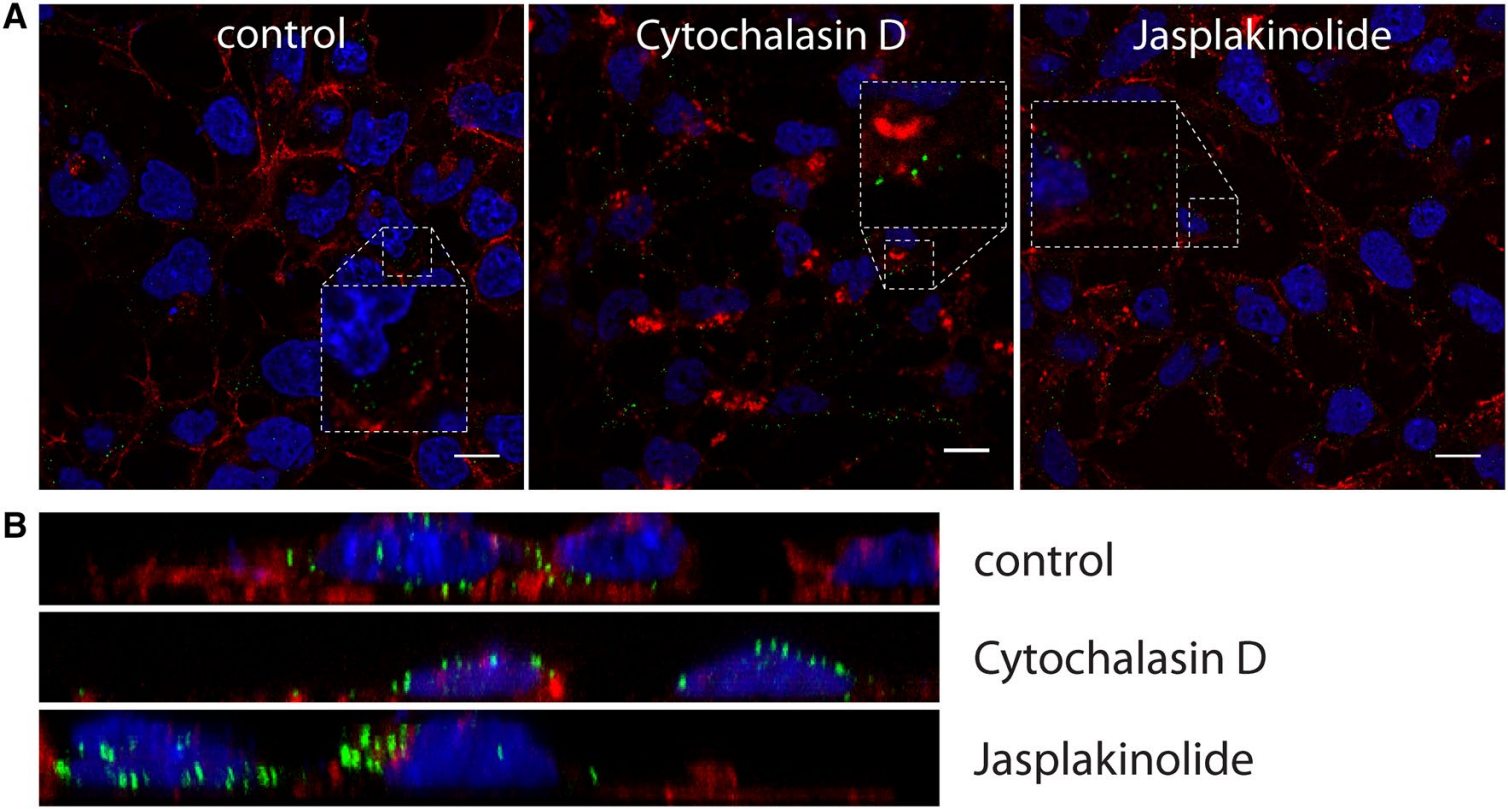
**Effect of cytoskeleton reorganization on CRCoV entry. A** Analysis of inhibitors effect on CRCoV entry, cells were pretreated with inhibitors, infected in presence of them and incubated for 1 h before they were washed off and fixed. CRCoV virions are presented in green, blue denotes DNA and red represents actin, each image is a single confocal plane while **B** presents maximum projection of their axial planes. Scale bar 10 µm.

The results so far suggest that CRCoV enters HRT-18G cells via caveolin- and dynamin-dependent endocytosis, and that it is likely that fusion occurs early after internalization. Therefore, we examined the role of the cytoskeleton during virus entry. First, we evaluated the effect of an actin-disrupting agent (cytochalasin D), an actin stabilizing compound (jasplakinolide), and an inducer of microtubule depolimerization (nocodazole) [46, 47]. Additional file 2 shows that, while microtubules are not important for virus infection, actin plays a central role during the early stages of CRCoV infection. Disrupting actin filaments led to a reduction (by 84.5% ± 8.9) in viral infection, although filament stabilization had no effect on virus yield. Lack of this effect after post-entry treatment (Additional file 2) imply that actin is required during early events of CRCoV infection. Studies of virus localization revealed that after treatment with cytochalasin D, multiple viral particles localize to actin aggregates (Figures 11A and B); this implies that an intact actin cytoskeleton plays a role in intracellular transport of viruses.

## Discussion

To propagate, viruses need to deliver their genetic material into the host cell. To do this, enveloped viruses fuse with the cellular membrane prior to ejecting genomic RNA or DNA into the cytoplasm. This may take place on the cell surface or after endocytosis. Here, we identified the pathway by which CRCoV enters human epithelial cells (HRT-18G) [8].

First, we asked whether CRCoV enters cells via endocytosis. Sensitivity to lysosomotropic agents is considered good evidence of endocytosis [48]; therefore, we treated cells with inhibitors of endosome acidification (bafilomycin A and ammonium chloride). Both compounds hampered virus infection, suggesting that CRCoV enters HRT-18G cells via endocytosis rather than via direct membrane fusion. However, inhibitory effect of inhibitors altering the endosomal pH was observed also on the replication stage. Similar phenomenon was previously reported for SARS-CoV [49]. It was suggested that these compounds interfere with glycosylation of the viral proteins and in such a way suppress their expression and assembly [49, 50]. Nonetheless, we decided to present the data on virus replication in supplementary material and focus on virus entry to the cell.

No single pathway of entry has been reported for *Coronaviridae*. Indeed, studies have identified clathrin-dependent, caveolin-dependent, clathrin-and-caveolin-independent, and endocytosis-independent routes (see Table 1). To identify the route of entry used by CRCoV, we utilized inhibitors of several different pathways (see Additional file 1). However, it is worth remembering that chemical inhibitors may affect other phases of virus replication by interfering with viral and cellular proteins (e.g., kinases, GTPases) [32, 51]. Indeed, we observed such non-specific interaction and consequently it was not possible to test the effect of chemical inhibitors on virus replication, as virus yields were reduced by almost all compounds affecting intracellular trafficking. To further address this issue, we employed complementary approaches: siRNA-mediated knockdown of caveolin-1 and analysis of co-localization of CRCoV viral particles with markers of different intracellular compartments. The results revealed that CRCoV enters the cell via the caveolin-1 dependent pathway; indeed, CRCoV particles co-localized with endosomes coated with caveolin-1, but not with clathrin or endophilin (Additional file 6). Furthermore, siRNA-mediated depletion of caveolin-1 inhibited virus entry.

**Table 1:** Entry pathways described for Coronaviruses

Virus	Genus	Entry route	Cells	References
HCoV-229E	Alpha	pH dependent endocytosis	Caco-2 (human colon adenocarcinoma), MRC-5 (human lungs)	[59]
		Caveolin-dependent endocytosis, lipid raft mediated	Human primary fibroblasts, L132 (human embryonic lung cell line)	[15]
		Endocytosis—laboratory strains, membrane fusion—clinical isolates	HeLa-229 (human cervix), HAE (human airway epithelium)	[18]
		Membrane fusion	HEK 293T (human embryonic kidney), Caco-2 (human colon adenocarcinoma)	[16]
HCoV-NL63	Alpha	Clathrin-dependent endocytosis	LLC-MK2 (rhesus monkey kidney) HAE (human airway epithelium)	[60]
Feline infectious peritonitis	Alpha	Clathrin- and caveolin-independent endocytosis	Primary feline monocytes	[14]
Transmissible gastroenteritis virus	Alpha	Lipid raft mediated	ST (swine testicles)	[61]
Canine enteric *Coronavirus*	Alpha	Lipid raft mediated	A72 (canine fibroma)	[62]
Mouse hepatitis virus	Beta	Cell membrane fusion	Mouse 3T3-L2 fibroblast	[63]
		pH dependent endocytosis	Murine fibroblast	[64]
		Lipid raft mediated	DBT cells (mouse astrocytoma)	[65]
SARS-CoV	Beta	Clathrin-dependent endocytosis	HepG2 (human hepatoma)	[13]
		Membrane fusion	Vero E6 (African green monkey kidney)	[66]
		Lipid raft mediated	Vero E6 (African green monkey kidney)	[11]
		Clathrin- and caveolin-independent endocytosis	HEK 293E (human embryonic kidney) , Vero E6 (African green monkey kidney)	[12]
		Membrane fusion	Vero E6 (African green monkey kidney), HEK 293T (human embryonic kidney)	[67]
MERS-CoV	Beta	Virus-cell fusion	HEK 293T (human embryonic kidney), Caco-2 (human colon adenocarcinoma)	[17]
HCoV-OC43	Beta	Caveolin-dependent endocytosis	HCT-8 (human adenocarcinoma)	[68]
Avian infectious bronchitis virus	Gamma	Low-pH-dependent virus-cell fusion	BHK (hamster kidney)	[69]
		Lipid raft mediated	Vero (African green monkey kidney)	[70]

Next, we focused on dynamin, an important GTPase involved in vesicle scission during endocytosis. While it is most commonly associated with clathrin-mediated endocytosis, it also plays a role in phagocytosis and caveolin-1, ILR-2-, and flotillin-dependent endocytosis [14, 40, 41]. To do this, we used three dynamin inhibitors, two that inhibit GTPase activity and one that blocks the lipid-binding domain of dynamin. The results led us to conclude that CRCoV entry into HRT-18G cells is dynamin-dependent.

In order to monitor intracellular trafficking of CRCoV and to determine the specific point of virus-cell membrane fusion, we examined co-localization of the CRCoV nucleocapsid protein and endosomal vesicle markers. The results showed that CRCoV was present in early endosomes but absent from late and recycling endosomes and lysosomes. This indicates that fusion of CRCoV and cellular membranes occurs at an early stage, before the endosome recruit Rab 7 and progresses to the late phase. This is different from the majority of coronaviruses, which travel far along the endocytic pathway to access high cathepsin activity (e.g., SARS-CoV, MHV, FIPV, porcine epidemic diarrhea coronavirus) [2, 28, 30]. The pathway used by CRCoV is more similar to that used by MERS-CoV reported to utilize furin as well as transmembrane protease serine 2 (TMPRSS2) [2, 17, 28, 29, 31]. We tested the influence of furin inhibitor decanoyl-RVKR-chloromethyl ketone in order to study the importance of furin for CRCoV entry. No inhibition of virus replication or cell to cell spread was noted, showing that furin processing is not relevant for CRCoV entry. One may however speculate that regardless of fusion with the host membrane occurring early after internalization, CRCoV may still utilize cathepsins similarly to most coronaviruses. Even if cathepsins is considered to require acidic pH for activation this dependency varies according to substrate and so far a few substrates were described to be cleaved in slightly acidic pH comparable to that achieved before endosome maturation [52–54]. Another potential explanation is cleavage by other proteases such as cell surface TMPRSS2 recently reported to be preferred over cathepsins by several coronaviruses [18, 29].

Our last objective was to study the role of the cytoskeleton during CRCoV infection. We discovered that in cells treated with cytochalasin D prior to infection, virus replication was reduced and during entry the virions accumulated on actin aggregates. Surprisingly, another actin inhibitor, jasplakinolide, had no significant effect on virus replication or entry. Interestingly, cytochalasin D and jasplakinolide affect the actin cytoskeleton via opposing mechanisms: cytochalasin D inhibits polymerization of actin subunits, whereas jasplakinolide stabilizes filaments by inhibiting depolymerization [46, 55]. The results obtained using these two agents suggest that actin filaments, but not actin reorganization, are required for CRCoV entry.

Coronaviruses are a family of viruses with high zoonotic potential. As such, they present a real threat to the global economy and to public health [4, 56–58]. Here, we show that CRCoV relies on dynamin-dependent, caveolin-1-mediated endocytosis to enter host cells. Moreover, we showed that fusion with the host membrane occurs early after internalization.

## Additional files

**Additional file 1.** **Inhibitors of endocytosis and their modes of action.** The table consists of a list of inhibitors used in this study with detailed information regarding their provider and applied concentration.

**Additional file 2.** **Chemical inhibitors effect on CRCoV infection.** Graphs shows number of virus positive cells at 5^th^ day pi normalized to control. HRT-18G cells were treated with acidification (A), dynamin (B), cell kinases (C), cytoskeleton (D), clathrin (E), macropinocytosis (F) and caveolin (G) inhibitors. Compounds were present prior and during the infection (white) or only after infection (gray). Cells were propagated in their presence until harvested.

**Additional file 3.** **Co-localization of CRCoV with markers of late endosomes and lysosomes.** A. CRCoV do not co-localize with late endosomes marker Rab7. B. CRCoV do not co-localize with lysosome marker LAMP1. C. Negative control D. Co-localization change in time. Cells treated with virus were synchronized on ice for 60 min and incubated at 37  °C before they were washed and fixed. Rab7 and LAMP1 are presented in red and CRCoV nucleocapsid protein in green. Cell nuclei are blue. Scale bar 10 µm.

**Additional file 4.** **CRCoV do not co-localize with recycling endosomes marker Rab11.** Cells treated with virus were synchronized on ice for 60 min and incubated at 37  °C before they were washed and fixed. Rab11 are presented in red and CRCoV nucleocapsid protein in green. Cell nuclei are blue. Scale bar 10 µm. Graph presents co-localization change in time.

**Additional file 5.** **Potential furin cleavage site prediction** Graphs show potential furin cleavage sites in the spike protein sequence of CRCoV isolate 4182 (**A, B**), K9 strain (**C**), K37 strain (**D**), K39 strain (**E**) and BJ232 strain (**F**).

**Additional file 6.** **CRCoV do not co-localize with endophilin.** Cells treated with virus were synchronized on ice for 60 min and incubated at 37  °C before they were washed and fixed. Endophilin are presented in red and CRCoV nucleocapsid protein in green. Cell nuclei are blue. Scale bar 10 µm. Graph presents co-localization change in time.

## Abbreviations

CRCoV: canine respiratory coronavirus
EEA1: early endosome antigen 1
MERS-CoV: Middle East respiratory syndrome coronavirus
HCoVs: human coronaviruses
SARS-CoV: severe acute respiratory syndrome coronavirus
CIRD: canine infectious respiratory disease
BCoV: bovine coronavirus
CECoV: canine enteric coronavirus
FIPV: feline infectious peritonitis virus
TCID_50_: tissue culture infectious dose
BSA: bovine serum albumin
CHT_x_B: cholera toxin subunit B
MβCD: methyl-β-cyclodextrin
EIPA: 5-(*N*-ethyl-*N*-isopropyl)amiloride
AMTD: 1-aminoadamantane
