# Supplementary material for: Canine respiratory coronavirus employs caveolin-1-mediated pathway for internalization to HRT-18G cells
Source: Vet Res. 2018 Jul 3;49:55. doi: 10.1186/s13567-018-0551-9 (PMC6029178; doi:10.1186/s13567-018-0551-9)
Supplement: Supplementary file 1 — Additional file 1. Inhibitors of endocytosis and their modes of action. The table consists of a list of inhibitors used in this study with detailed information regarding their provider and applied concentration. [file 13567_2018_551_MOESM1_ESM.docx]

| **Compound** | **Effect** | **Concentration** | **Provider** |
| --- | --- | --- | --- |
| Amantadine (1-aminoadamantane) | Inhibits clathrin-mediated endocytosis by stabilization of clathrin-coated pits | 500 µM | Sigma-Aldrich Poland |
| Pitstop 2 | Competitively inhibits clathrin terminal domain (TD) | 5 µM | Abcam |
| Chlorpromazine | Inhibits clathrin-mediated endocytosis, loss of clathrin and the AP2 adaptor complexes from cell surface | 5 µM | Sigma-Aldrich Poland |
| Filipin III | Alters membrane permeability and associated functions by binding to membrane sterols | 1 μg/mL | Sigma-Aldrich Poland |
| Nystatin | Sterol-binding agent that disassembles caveolae | 50 µg/mL | Sigma-Aldrich Poland |
| MβCD (Methyl-β-cyclodextrin) | Extracts cholesterol from lipid membranes | 2 mM | Sigma-Aldrich Poland |
| EIPA (5-(N-ethyl-N-isopropyl)amiloride) | Blocker of Na^+^/H^+^ antiport | 10 µM | Sigma-Aldrich Poland |
| IPA-3 | Inhibitor of group I p21-activated kinase (PAK1) | 20 µM | Sigma-Aldrich Poland |
| Wortmanin | Inhibits phosphoinositide-3-kinase (PI3K) | 5 µM | Calbiochem |
| Dynasore | Interferes with the GTPase activity of dynamin1, dynamin2, and Drp1 (mitochondrial dynamin) | 40 µM | Abcam |
| Iminodyn 22 | Broad spectrum dynamin inhibitor, Binds to the GTPase domain at an allosteric site and displays uncompetitive antagonism with respect to GTP | 15 µM | Abcam |
| Mitmab | Cell-permeable dynamin I and dynamin II inhibitor, targets the pleckstrin homology (PH) (lipid binding) domain | 5 µM | Abcam |
| NH_4_Cl | Increases the pH of endosomes | 50 mM | Bioshop |
| Bafilomycin A1 | Inhibitor of vacuolar type H^+^-ATPase, prevents acidification of endosomes | 10 nM | Sigma-Aldrich Poland |
| Cytochalasin D | Disrupts actin polymerization | 10 µM | Sigma-Aldrich Poland |
| Jasplakinolide | Stabilizes actin microfilaments | 150 nM | Calbiochem |
| Nocodazole | Depolymerizes microtubules | 0,5 μM | Sigma-Aldrich Poland |
| cell permeable Rho inhibitor CT04 | Selectively inactivate the GTPases RhoA, RhoB, and RhoC | 1 µg/mL | Cytoskeleton Inc |
| NSC23766 trihydrochloride | inhibitor of Rac1, a Rho-family GTPase | 100 μM | Sigma-Aldrich Poland |
| Y27632 ((*R*)-(+)-*trans*-4-(1-Aminoethyl)-N-(4-Pyridyl)cyclohexanecarboxamide dihydrochloride) | ROCK (Rho-associated coiled coil forming protein serine/threonine kinase) inhibitor | 10 μM | Sigma-Aldrich Poland |
| Decanoyl-Arg-Val-Lys-Arg-chloromethyl ketone | Blocks activity of all seven convertases (PC1, PC2, PC4, PACE4, PC5, PC7 and furin) | 5-100 μM | Santa Cruz Biotechnology |

**Additional file 1 Inhibitors of endocytosis and their modes of action**
