# Supplementary figures and images for: Canine respiratory coronavirus employs caveolin-1-mediated pathway for internalization to HRT-18G cells
Source: Vet Res. 2018 Jul 3;49:55. doi: 10.1186/s13567-018-0551-9 (PMC6029178; doi:10.1186/s13567-018-0551-9)

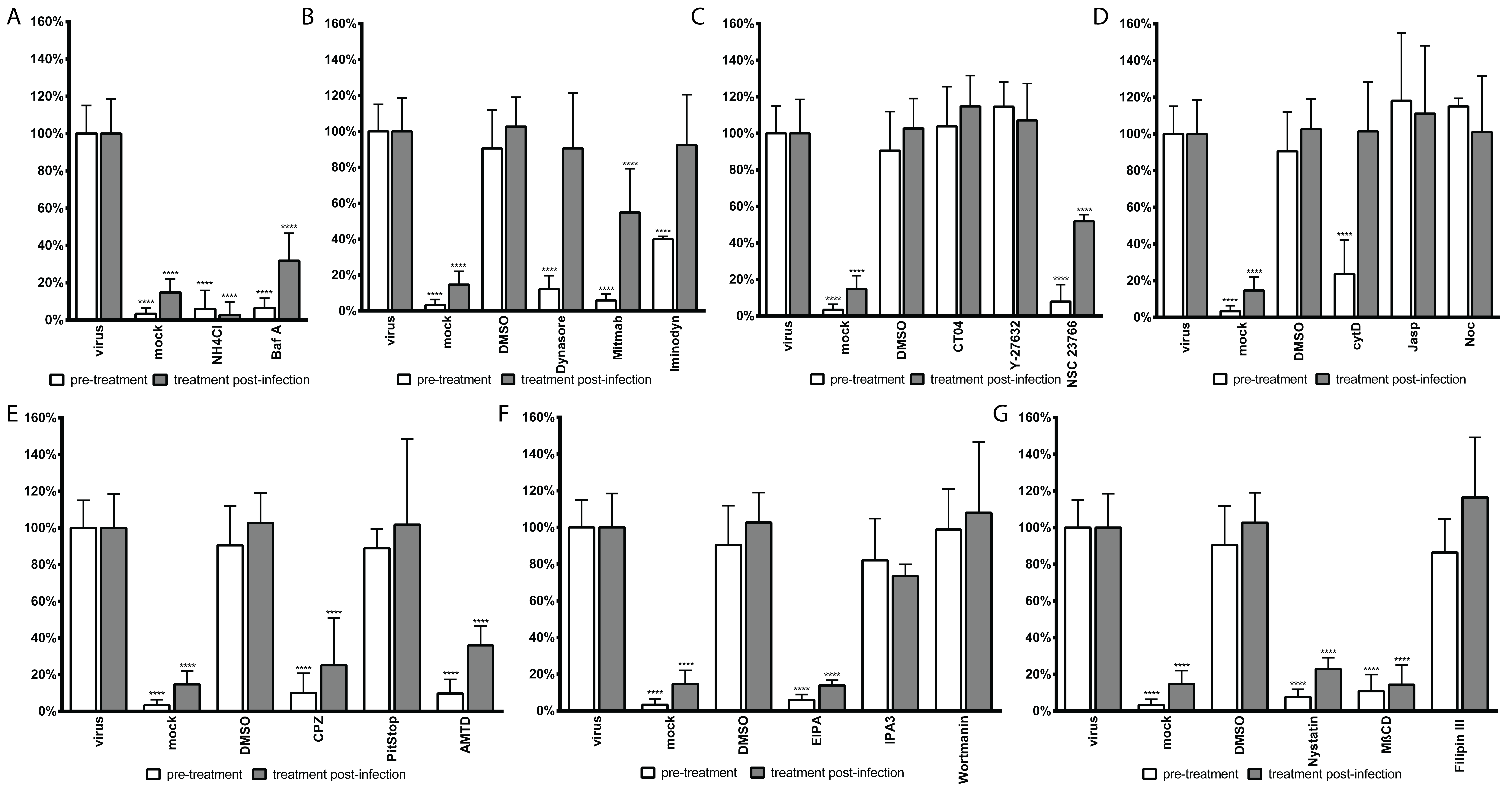

Supplement: Supplementary file 2 — Additional file 2. Chemical inhibitors effect on CRCoV infection. Graphs shows number of virus positive cells at 5th day pi normalized to control. HRT-18G cells were treated with acidification (A), dynamin (B), cell kinases (C), cytoskeleton (D), clathrin (E), macropinocytosis (F) and caveolin (G) inhibitors. Compounds were present prior and during the infection (white) or only after infection (gray). Cells were propagated in their presence until harvested. [file 13567_2018_551_MOESM2_ESM.tif]

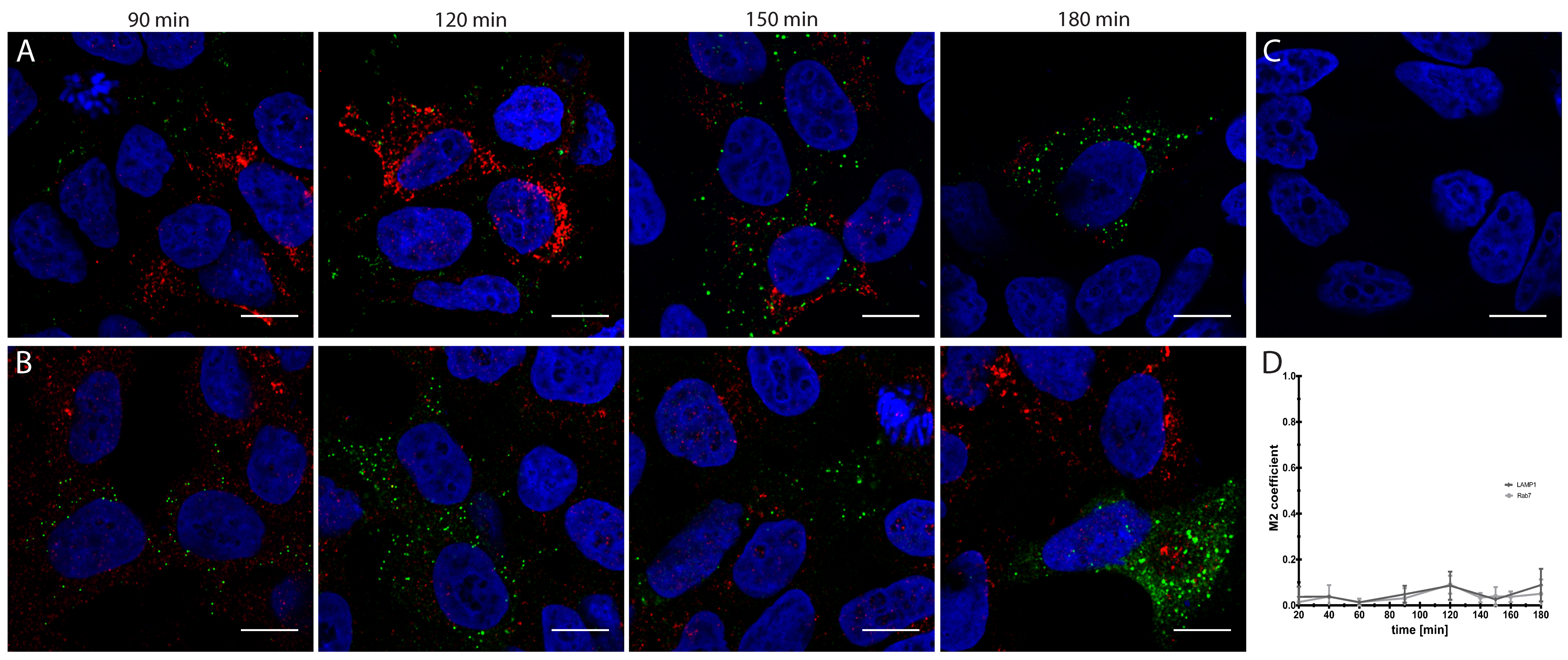

Supplement: Supplementary file 3 — Additional file 3. Co-localization of CRCoV with markers of late endosomes and lysosomes. A. CRCoV do not co-localize with late endosomes marker Rab7. B. CRCoV do not co-localize with lysosome marker LAMP1. C. Negative control D. Co-localization change in time. Cells treated with virus were synchronized on ice for 60 min and incubated at 37 °C before they were washed and fixed. Rab7 and LAMP1 are presented in red and CRCoV nucleocapsid protein in green. Cell nuclei are blue. Scale bar 10 µm. [file 13567_2018_551_MOESM3_ESM.tif]

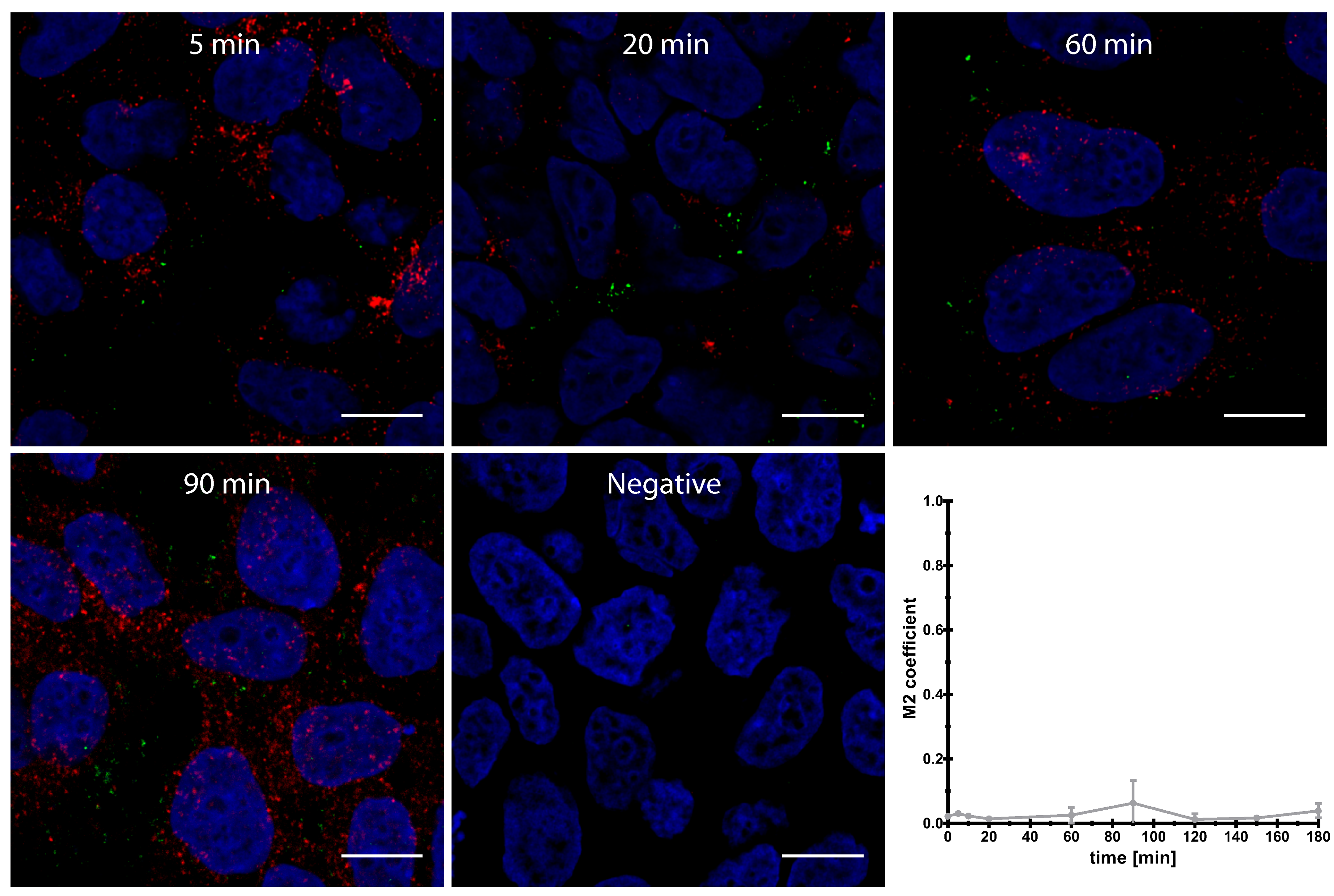

Supplement: Supplementary file 4 — Additional file 4. CRCoV do not co-localize with recycling endosomes marker Rab11. Cells treated with virus were synchronized on ice for 60 min and incubated at 37 °C before they were washed and fixed. Rab11 are presented in red and CRCoV nucleocapsid protein in green. Cell nuclei are blue. Scale bar 10 µm. Graph presents co-localization change in time. [file 13567_2018_551_MOESM4_ESM.tif]

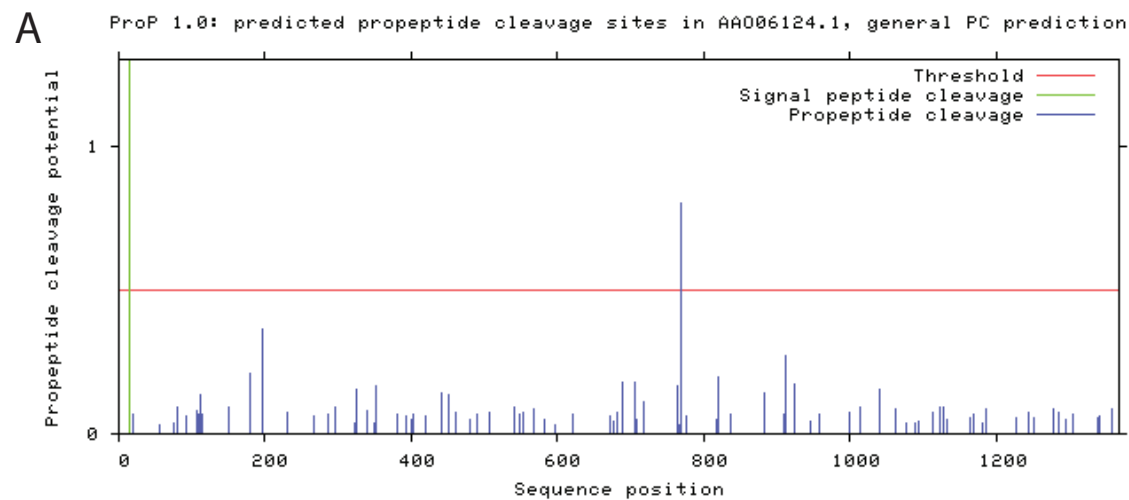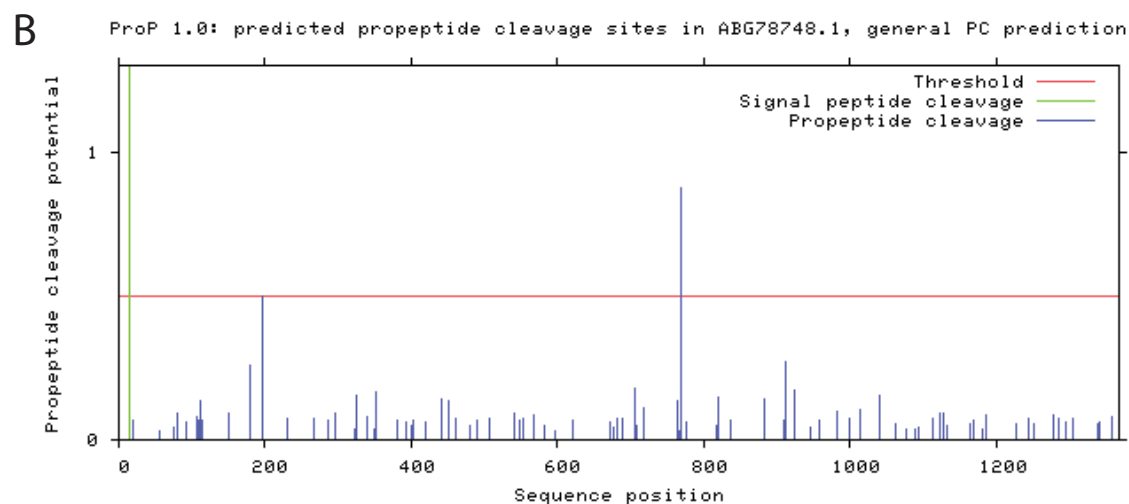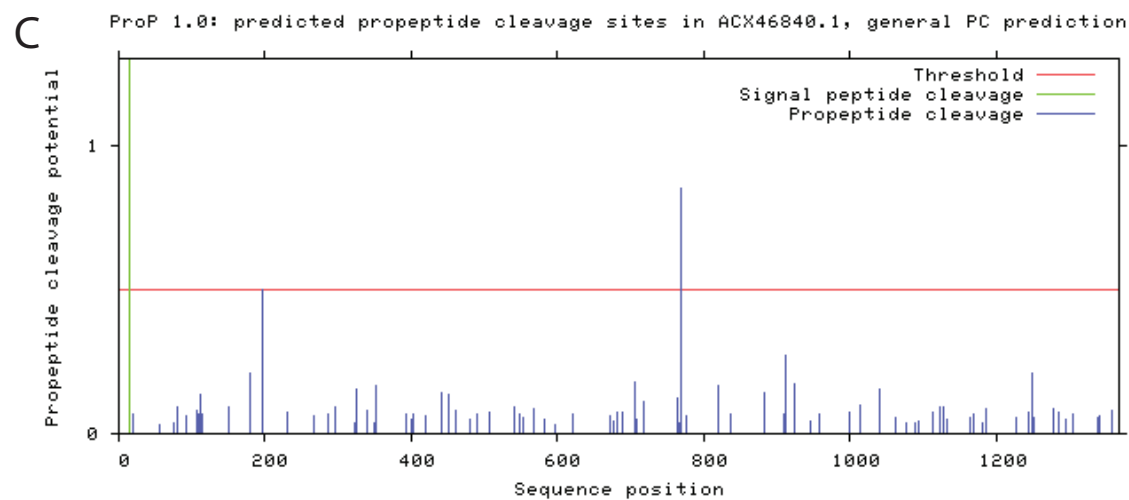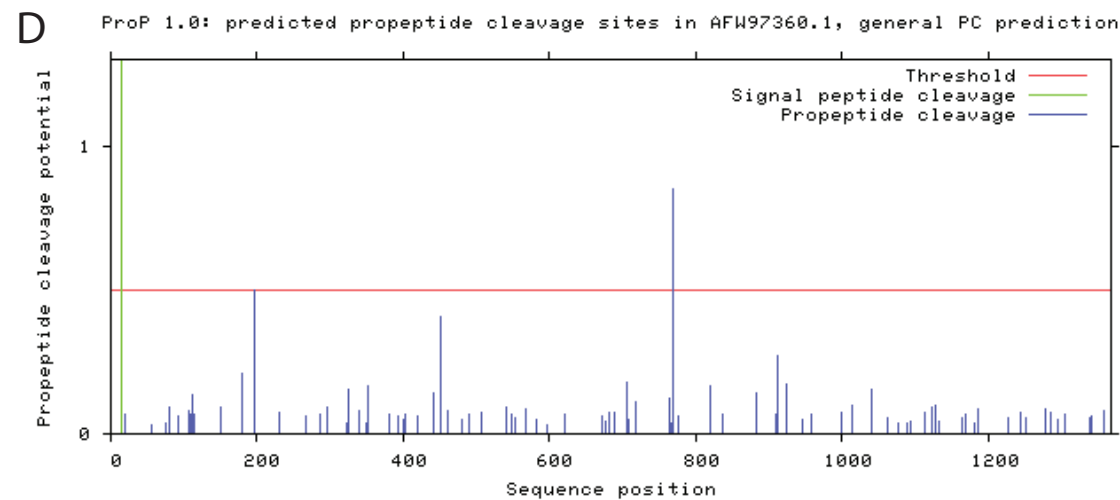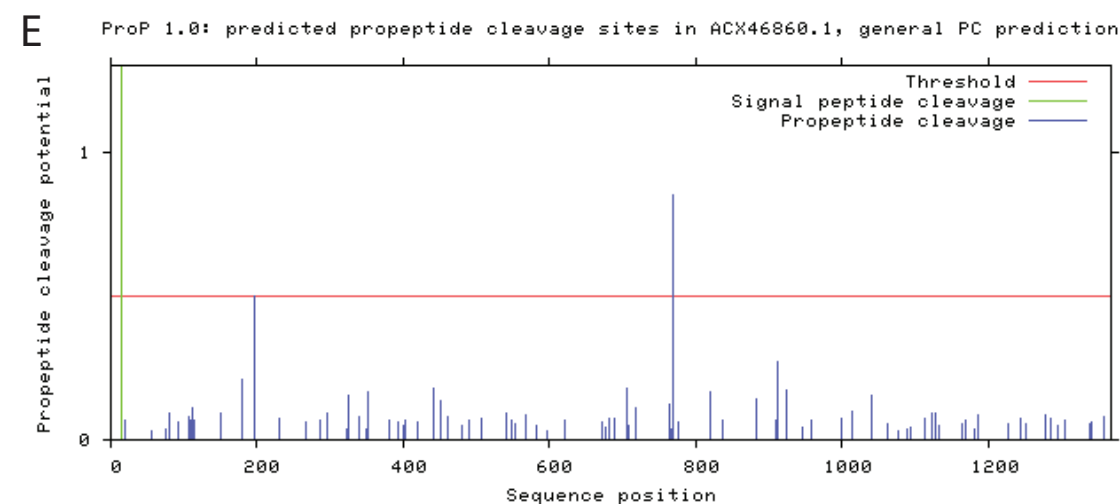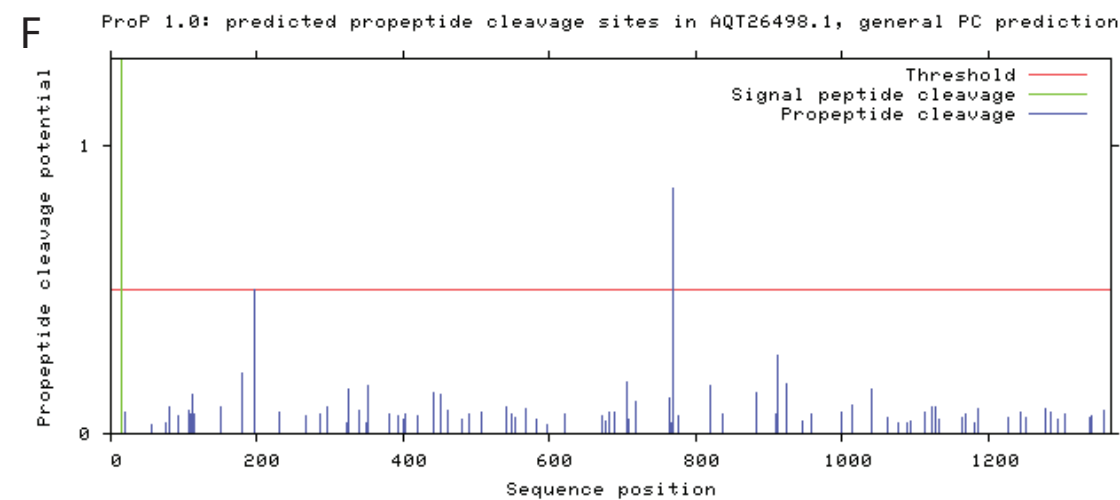

Supplement: Supplementary file 5 — Additional file 5. Potential furin cleavage site prediction Graphs show potential furin cleavage sites in the spike protein sequence of CRCoV isolate 4182 (A, B), K9 strain (C), K37 strain (D), K39 strain (E) and BJ232 strain (F). [file 13567_2018_551_MOESM5_ESM.pdf]

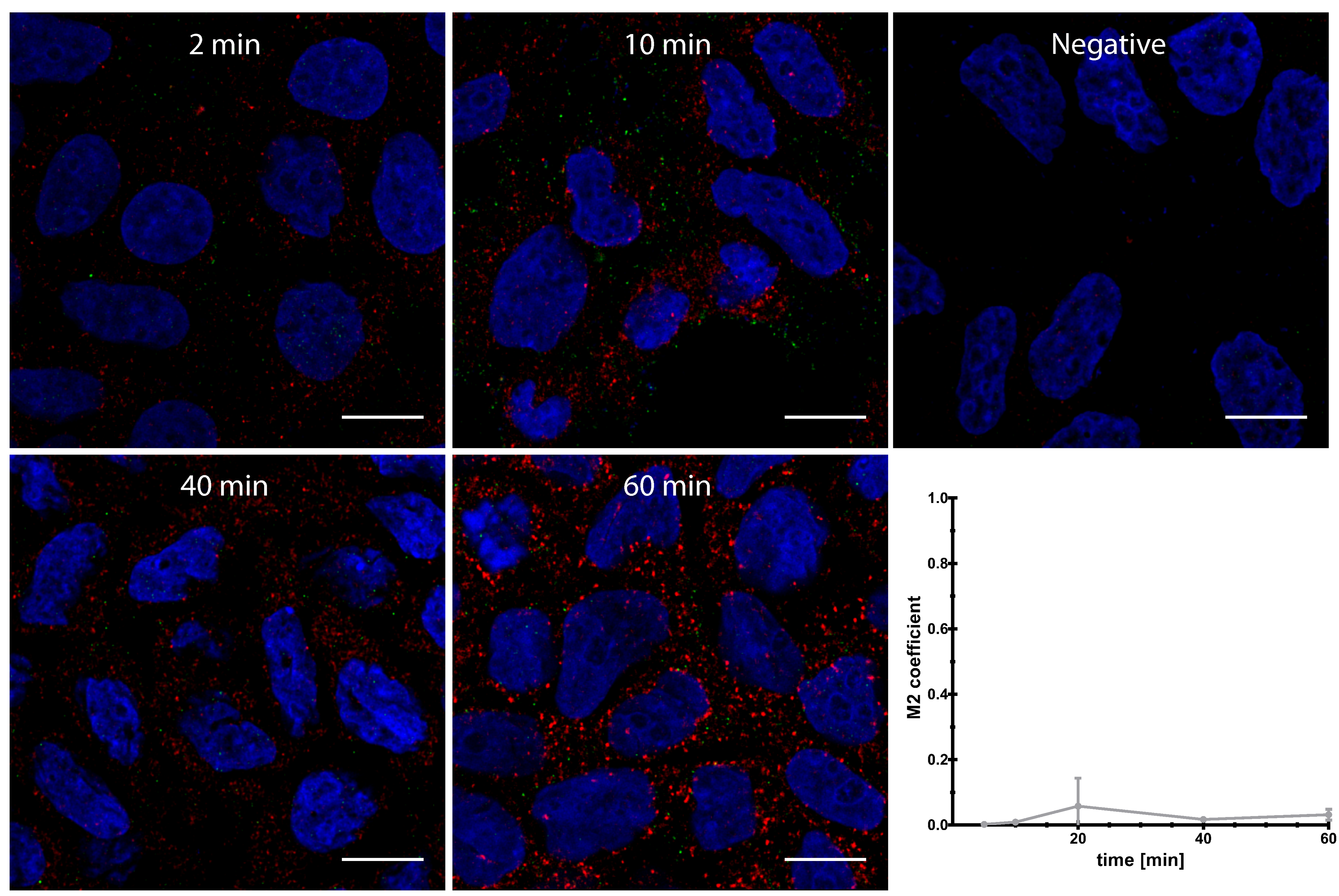

Supplement: Supplementary file 6 — Additional file 6. CRCoV do not co-localize with endophilin. Cells treated with virus were synchronized on ice for 60 min and incubated at 37 °C before they were washed and fixed. Endophilin are presented in red and CRCoV nucleocapsid protein in green. Cell nuclei are blue. Scale bar 10 µm. Graph presents co-localization change in time. [file 13567_2018_551_MOESM6_ESM.tif]
